# Sarcopenia as a prognostic marker in patients undergoing pancreaticoduodenectomy: an updated meta-analysis

**DOI:** 10.3389/fonc.2025.1656834

**Published:** 2025-09-29

**Authors:** Jie He, Jia Li, Jia Liu, Meng Liu

**Affiliations:** ^1^ School of Clinical Medicine, Chengdu Medical College, Chengdu, Sichuan, China; ^2^ Department of Pulmonary and Critical Care Medicine, The First Affiliated Hospital of Chengdu Medical College, Chengdu, Sichuan, China; ^3^ Key Laboratory of Geriatric Respiratory Diseases of Sichuan Higher Education Institutes, Chengdu, Sichuan, China; ^4^ General Practice Department, The First Affiliated Hospital of Chengdu Medical College, Chengdu, Sichuan, China; ^5^ Department of Pulmonary and Critical Care Medicine, The First People’s Hospital of Chengdu City, Chengdu, Sichuan, China

**Keywords:** pancreaticoduodenectomy, sarcopenia, postoperative complications, disease-free survival, overall survival, meta-analysis

## Abstract

**Background:**

Sarcopenia is prevalent among patients undergoing pancreaticoduodenectomy (PD). However, the effect of sarcopenia on postoperative complications and the prognosis of patients undergoing PD remain controversial. This meta-analysis aimed to evaluate the potential use of sarcopenia as a prognostic indicator in patients undergoing PD.

**Methods:**

A systematic search was conducted using the databases of Web of Science, EMBASE, China National Knowledge Infrastructure, Cochrane Library, and PubMed from inception to March 14, 2025, to identify studies on sarcopenia in patients undergoing PD. The pooled prevalence of sarcopenia and its 95% confidence interval (CI) were calculated, and heterogeneity was assessed using the I² test. Associations between sarcopenia and major postoperative complications, postoperative pancreatic fistula (POPF), postoperative biliary fistula (POBF), mortality, disease-free survival (DFS), and overall survival (OS) were expressed as odds ratios (ORs) or hazard ratios (HRs) with 95% CIs. Statistical analyses were performed using Stata version 11.0.

**Results:**

This meta-analysis included 30 articles involving 5,323 participants. The prevalence of sarcopenia before PD was 35%. Patients with sarcopenia exhibited a significantly higher risk of major complications (Clavien–Dindo [CD] grade ≥ III) (OR = 1.84, 95% CI = 1.26–2.69, *P* = 0.002), POPF (OR = 1.47, 95% CI = 1.13–1.93, *P* = 0.004), and POBF (OR = 1.53, 95% CI = 1.05–2.25, *P* = 0.028) than those without sarcopenia. In addition, postoperative mortality was higher in patients with sarcopenia (OR = 3.52, 95% CI = 2.01–6.19, *P* = 0.002). Patients without sarcopenia exhibited better DFS and OS after PD than those with sarcopenia (DFS: HR = 2.28, 95% CI = 1.18–2.88, *P* < 0.001; OS: HR = 3.15, 95% CI = 2.49–3.98, *P* < 0.001).

**Conclusion:**

A high proportion of patients presented with sarcopenia before undergoing PD. Patients undergoing PD with sarcopenia face a higher risk of overall incidence of major complications (CD grade ≥ III), POPF, POBF, and mortality, and they exhibit worse DFS and OS than those without sarcopenia. Future studies should adopt stricter definitions of sarcopenia to further validate these findings.

**Systematic review registration:**

https://www.crd.york.ac.uk/PROSPERO/view/CRD42025635939, identifier CRD42025635939.

## 1 Introduction

Pancreaticoduodenectomy (PD) is a complex surgical procedure for treating benign and malignant diseases in the pancreatic head, periampullary region, and distal common bile duct (1). The procedure involves the resection of the affected pancreatic tissue, along with segments of the duodenum, common bile duct, gallbladder, and portions of the stomach (2). Despite advancements in surgical approaches and perioperative management, PD remains a technically challenging and high-risk procedure. The postoperative complication rates of PD range from 30%–50% (3), emphasizing the necessity of identifying key risk factors.

Recent studies have highlighted the significant effect of sarcopenia on the clinical outcomes and prognosis of patients undergoing major surgeries (4). Sarcopenia is characterized by the progressive loss of skeletal muscle mass and is often accompanied by diminished muscle strength and an impaired capacity to perform daily activities (5, 6). Affected individuals typically experience reduced mobility, lower quality of life, and higher risk of adverse outcomes such as falls and mortality (7, 8). Contributing factors to sarcopenia include malnutrition, hormonal changes, chronic inflammation, alteration in gut microbiota, physical inactivity, and genetic and psychosocial influences (9–11). This condition is prevalent among older patients (12, 13) and is associated with a poor prognosis across various cancer types (14, 15). Sarcopenia is more common in patients undergoing PD. Balcer (16) reported that 49% of patients undergoing PD exhibited sarcopenia, with 10% diagnosed with sarcopenic obesity. Patients with sarcopenia often present with low body mass index (BMI), low skeletal muscle index (SMI), and reduced subcutaneous fat. The SMI at the third lumbar vertebra, derived from computed tomography (CT), is a reliable indicator of sarcopenia (17). For patients undergoing PD, routine CT scans are valuable for assessing tumor lesions and monitoring metastasis and for evaluating skeletal muscle mass without the need for additional radiation exposure.

However, the effect of comorbid sarcopenia on clinical outcomes and prognosis after PD remains unclear. Previous meta-analyses have identified sarcopenia as a prevalent comorbidity in patients undergoing PD, with those exhibiting preoperative sarcopenia experiencing higher morbidity, higher mortality, and poorer prognosis (18). Although several studies have investigated the association between sarcopenia and complications in patients undergoing PD, their findings remain inconclusive. This study aimed to evaluate the effect of sarcopenia on postoperative outcomes in patients undergoing PD and to provide a robust evidence base to inform perioperative management strategies.

## 2 Methods

### 2.1 Literature search strategy

This study adhered to the updated Preferred Reporting Items for Systematic Reviews and Meta-Analyses (2020) guidelines, and the protocol was registered with PROSPERO (CRD42025635939). The literature search, conducted by Jie He and Jia Liu, utilized the PubMed, Web of Science, Cochrane Library, China National Knowledge Infrastructure, WanFang, and Embase databases. The search spanned from the inception of the databases to March 14, 2025, and included only articles published in Chinese and English. Key search terms included “sarcopenia,” “frailty,” “muscle weakness,” “muscle atrophy,” “pancreaticoduodenectomy,” “Whipple procedure,” “pancreaticoduodenectomies,” “duodenopancreatectomy,” and “pancreatoduodenectomy.” Additionally, the references cited within the identified articles were reviewed. The search strategies employed across all databases were outlined.

### 2.2 Eligibility criteria

The inclusion criteria were as follows: (1) observational design, including cross-sectional, case–control, and cohort studies, regardless of sample size; (2) studies that diagnosed sarcopenia and PD using validated methods, defining sarcopenia as reduced muscle mass and strength with low physical performance; and (3) a study population comprising individuals who underwent PD. Included studies were required to provide access to the full text and allow for accurate data extraction. The exclusion criteria encompassed reviews, systematic reviews, case reports, commentaries, non-clinical trials, and duplicate publications based on the same cohort. Furthermore, studies lacking critical clinical data or outcome measures, or those exhibiting substantial risk of bias, were excluded.

### 2.3 Data extraction

The study data were independently extracted by two authors (Jie He and Jia Liu), and discrepancies were resolved through discussions. If consensus could not be reached, a third investigator adjudicated the issue. Key extracted parameters included baseline information (first author, country, publication date, study duration, study design, sample size, mean age, disease type, BMI, diagnostic criteria, and sarcopenia prevalence) and clinical outcome measures (Clavien–Dindo [CD] grade ≥ III complications, grade B/C postoperative pancreatic fistula [POPF], postoperative biliary fistula [POBF], mortality, disease-free survival [DFS], and overall survival [OS]) (19). Continuous variables were summarized as means and standard deviations (SDs); for studies reporting medians or ranges, means and SDs deviations were estimated using Hozo’s method (20).

### 2.4 Literature quality assessment

Study quality was independently assessed by at least two authors (Meng Liu and Jie He) by using standardized assessment tools. The risk of bias in the included studies was assessed with the Joanna Briggs Institute’s critical appraisal checklist (**Supplementary Table 1**). Prognostic studies were assessed using the Quality in Prognostic Studies (QUIPS) tool (21), which evaluates risk of bias across six key areas: selection bias, attrition bias, measurement bias of prognostic factors, measurement bias of outcomes, confounding factors, and bias related to statistical analysis and result presentation. The QUIPS tool was selected as the most suitable method for assessing the quality of the studies under review. We slightly modified the original tool by introducing the “not applicable” option for rating items in the bias domains. We employed three rating levels, namely, high, moderate, and low, to evaluate the risk of bias in each domain. A study was deemed to have a high or moderate risk of bias if any domain received a high or moderate rating. Conversely, a study was considered to have a low risk of bias if all six domains were rated as low risk. Disagreements during quality assessment were addressed through discussions by the reviewers (Jia Li and Jia Liu) or resolved by expert arbitration (Jiaqing Jiang) when necessary.

### 2.5 Outcome measures

The study aimed to: (1) examine the sarcopenia prevalence in patients undergoing PD; (2) examine the association between sarcopenia and key complications, including pancreatic fistula, biliary fistula, and mortality in patients undergoing PD; (3) investigate the effect of sarcopenia on the prognosis of patients undergoing PD.

### 2.6 Statistical analysis

RevMan version 5.3.5 and Stata version 11.0 (Cochrane Collaboration, Oxford, UK) were utilized for the meta-analysis. Sarcopenia prevalence was calculated using raw data or reported prevalence (%). In longitudinal studies reporting prevalence at multiple time points, the overall prevalence for a specific period was used. A meta-analysis of prevalence was conducted using a generalized linear mixed model with a logit transformation and a fixed or random effects model. The relationships between sarcopenia occurrence and PD, and its effects on mortality and complications, were evaluated using adjusted odds ratios (ORs) with 95% confidence intervals (CIs) and adjusted hazard ratios (HRs) with 95% CIs, respectively. Heterogeneity was assessed using the I² statistic and Cochran’s Q test within random effects models. Intra-study heterogeneity was estimated via restricted maximum likelihood estimation, with significance determined by the Q value, which indicates whether moderator exploration is required, and the I² statistic, which quantifies the percentage of total variability attributable to heterogeneity (none: < 25%; low: 25%–50%; moderate: 51%–75%; high: ≥ 75%).

Subgroup analyses were conducted to identify the factors contributing to heterogeneity, including race and sarcopenia definition criteria. Publication bias was assessed using Egger’s test, Begg’s test, and funnel plots. A sensitivity analysis based on the leave-one-out approach was planned if a sufficient number of studies were available for evaluating the robustness of the findings. Statistical significance was set at a two-tailed *P <* 0.05.

## 3 Results

### 3.1 Eligible studies

**Figure 1:**
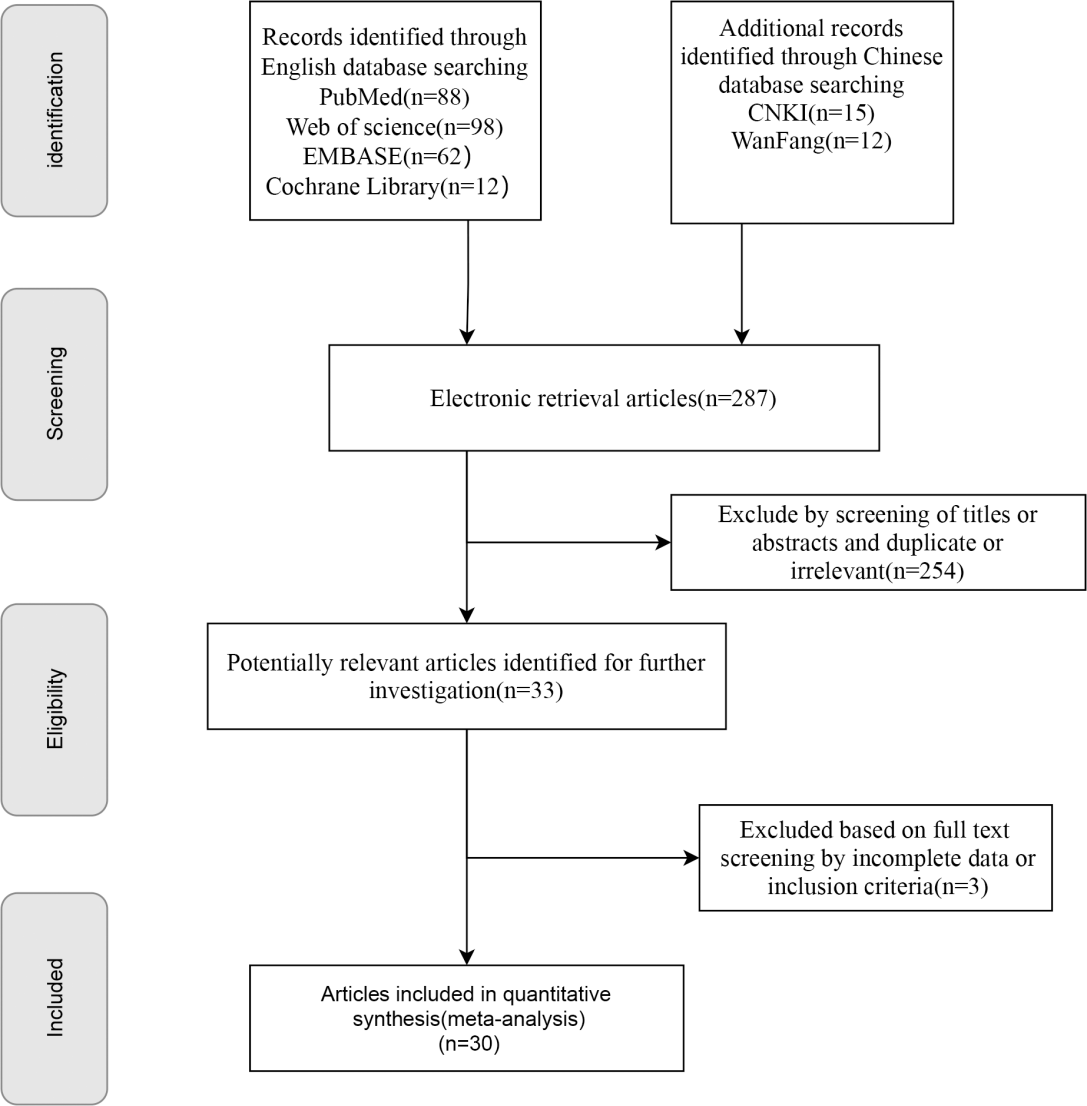
Flow diagram of literature screening.

A total of 287 publications were retrieved. After multiple rounds of screening, 30 studies were included. The initial search yielded 287 articles, which were narrowed to 254 articles after removing duplicates; among these, 33 were selected for further analysis based on their titles and abstracts. The full texts of 33 articles were reviewed, resulting in the exclusion of three articles for the reasons outlined in **Figure 1**. Additional irrelevant or duplicate studies were excluded, leaving 30 articles that met the inclusion criteria (16, 22–50); among these, 28 examined the prevalence of sarcopenia in patients undergoing PD (16, 22–26, 29–45, 47–51), five investigated sarcopenic obesity (16, 27, 29, 33, 40), 19 focused on major complications (16, 23–27, 29–31, 34–36, 38–40, 44–46, 49), 11 addressed postoperative mortality (16, 23, 24, 30, 31, 36, 41, 42, 44, 47, 49), 17 explored POPF (26–32, 34, 36, 38, 41, 43–45, 47–49), three studies reported the differences in SMI between patients with and without POPF (28, 33, 34), eight examined POBF (26, 29, 30, 32, 34, 41, 43, 48), five reported on the relationship between sarcopenia and DFS (16, 25, 26, 30, 38), and six analyzed the association between sarcopenia and OS in patients undergoing PD (16, 25, 26, 30, 38, 40). All included studies were cohort studies. The screening details are presented in **Figure 1**, basic information on the included studies is presented in **Table 1** and **Supplementary Table 2**, and the quality assessment is provided in **Supplementary Tables 3** and **4**.

### 3.2 Characteristics of the included articles

**Table 1** and **Supplementary Table 2** present the characteristics of the included articles. A total of 30 studies involving 5,323 patients were included. The age of the participants ranged from 27 to 88 years. Geographically, 18 studies were conducted in Asia, 8 in Europe, and 3 in North America. Eighteen articles used SMI to define sarcopenia, seven articles used the psoas muscle index (PMI)A to define sarcopenia, and five articles employed other indicators to define sarcopenia. Among these studies, 2 were prospective, and 26 were retrospective. Muscle mass was assessed using dual-energy X-ray absorptiometry, bioelectrical impedance analysis, or CT, whereas muscle strength was measured using a hand dynamometer (**Table 1**). Physical performance was evaluated based on gait speed, measured through 4-, 5-, and 6-minute walk tests. The quality assessment is presented in **Supplementary Tables 3** and **4**.

**Table 1:** Demographic characteristics of included studies.

Study	Year	Case	n	Male	Country	Age(years)	Guideline used	Sarcopenia measures	Inspection equipment
Xu Z	2024	68	207	125	China	33-79	Sarcopenia was defined as an SMI <53.00 cm²/m² for males with a BMI≥25 kg/m², <43.00 cm²/m²for males with a BMI<25 kg/m², and <41.00 cm²/m² for females	SMI	CT
Wielsoe S	2024	16	122	87	Denmark	67±9	EWGSOP	SMI, Handgrip strength	CT
Utsumi M	2024	24	80	40	Japan	71±8.5	The cut-off values for PMI were 5.50 and 4.49 cm²/m² in men and women	PMI	CT
Qu G	2024	83	162	92	China	63.78±10.27	Japanese Society of Hepatology	SMI	CT
Guarneri G	2024	297	371	202	Italy	60-74	SO was defined, in line with previous literature, as a high ratio between VFA/TAMA, specifically VFA/TAMA ratio greater than 3.2	SMI	CT
Balcer K	2024	94	196	108	France	47-67	obese (BMI>30 kg/m²) women with SMI<38.5 cm²/m², non-obese (BMI<30 kg/m²) women with SMI<32 cm²/m², obese men with SMI<52.4 cm²/m², and non-obese men with SMI<42 cm²/m²	SMI	CT
Tazeoglu D	2023	83	179	105	Turkey	60.45±13.08	PMI was calculated with the formula (right psoas area left psoas area)/height squared (m²). The cut-off value for PMI sarcopenia was ≤5.3 for males and≤3.6 for females	Psoas muscular index	CT
Takagi K	2023	29	110	63	Japan	46-86	They defined sarcopenia using sex-specific cutoff values of PMI, which were 6.36 cm²/m² for men and 3.92 cm²/m² for women	Psoas muscular index	CT
La Vaccara V	2023	30	82	50	Italy	None	males <55,4 cm²/m²and females < 38,9 cm²/m².	SMI	CT
Hayashi H	2023	67	169	105	Japan	30-92	the international consensus of a SMI of <52.4 cm²/m²for men and <38.9 cm²/m² for women.	SMI	CT
Cai Z	2023	47	129	78	China	62.4±12.1	Sex-specific SMI cut-off values of 42.2 cm²/m² for men and 33.9 cm²/m² for women were used to define sarcopenia	SMI	CT
Umezawa S	2022	44	88	65	Japan	68-78	PMI(cm²/m²):6.36≦Male, 3.98≦Female	PMI	CT
Nauheim DO	2022	83	333	161	USA	68.5±11.1	AWGS	PMI	CT and MRI
Maekawa T	2022	41	164	104	Japan	62-76	cut-off values: L3 SMI, <40.5 cm²/m² for men and <33.5 cm²/m² for women	SMI	CT
Sui K	2017	87	354	203	Japan	70±11	AWGS	SMI	CT and BIA
Aoki Y	2022	19	180	102	Japan	66-80	EWGSOP2	SMI, HS, GS	Dual-energy x-ray absorptiometry
Pessia B	2021	32	68	not available	Italy	62.5	L3 skeletal muscle index ≤38.5 cm²/m² for women and ≤52.4 cm²/m² for men	SMI	CT
Peng YC	2021	20	116	68	Taiwan	66.2±11.9	Sex-specific cutoff values for sarcopenia were determined as 42.2 cm²/m² for men and 33.9 cm²/m² for women,	SMI	CT
Duan K	2021	108	265	136	China	59.5±13.9	The cutoff value of SMI was 47.32 cm²/m² for male and 40.65 cm²/m² for female patients	SMI	CT
Xu JY	2020	59	152	89	China	63.2±11.6	4.78 cm²/m² for male patients and 3.46 cm²/m² for female	PMI	CT
Centonze L	2020	36	110	48	Italy	59-75	The lowest quartile TPA threshold for men was 492 mm²/m² versus 362 mm²/m² for women	SMI, HS	CT and MRI
Umetsu S	2018	48	65	47	Japan	31-81	The cut-off values for PMI in males and females were 5.93 and 3.54 cm²/m²	PMI	CT
Tankel J	2018	16	61	32	Israel	71±8.5	For male patients this was 83.41 cm²/m² and for females 65.28 cm²/m²	Total psoas muscle area	CT
Stretch C	2018	50	123	71	Canada	68.5±10.8	SMI for each sex (<47.7 cm²/m² for males and <36.5 cm²/m² for females)	SMI	CT
Takagi K	2017	55	219	143	Japan	65.9±11.7	The cut-off values for the lowest quartiles of SBI were 68.5 cm²/m² for men and 52.5 cm²/m² for women.	SMA/BSA index	CT
Sandini M	2016	30	124	63	Italy	65.5–76.8	The cutoff values of TAMA<41 cm²/m² for females and of TAMA<43 (with BMI <25 kg/m²) or <53 (with BMI ≥25) for males	total abdominal muscle area	CT
Nishida Y	2016	132	266	181	Japan	27-87	sarcopenia is defined as a skeletal muscle index (SMI) = ([skeletal muscle area at L3]/[height]2)20 of <43 cm²/m² in men with a BMI of<25 kg/m², <53 cm²/m² in men with a BMI of ≥25 kg2/m², and <41 cm²/m² in women.	SMI	CT
Peng P(Men)	2012	74	296	296	USA	65.2±10.8	The lowest quartile TPA threshold for men was 492 mm²/m² versus 362 mm²/m² for women.	Total psoas muscle area	CT
Peng P(Women)	2012	65	261	0	USA	66.3±10.3	The lowest quartile TPA threshold for men was 492 mm²/m² versus 362 mm²/m² for women.	Total psoas muscle area	CT
Nakajima T	2024	NA	153	78	Japan	44-88	AWGS	SMI, HS	CT
Phillips ME	2024	57	118	NA	UK	65.1±10.5	SO was defined as those with a low skeletal muscle index and a BMI > 30 kg/m^2^ or as a ratio of VFA/SMI with a cut-off of 2.5 m^2^	SMI, HS	CT

SO, Sarcopenic obesity; VFA, visceral fat area; TAMA, total abdominal muscle area; SMI, skeletal muscle index; PMI, psoas muscular index; BMI, body mass index; TPA, total psoas area; EWGSOP, European Working Group on Sarcopenia in Older People; AWGS, Asian Working Group for Sarcopenia; L3, third lumbar vertebra level; SBI, sarcopenic obesity; HS, handgrip strength; GS, gait speed; SMA/BSA, skeletal muscle area/body surface area; CT, computed tomography; MRI, magnetic resonance imaging; BIA, bioelectrical impedance analysis; NA, not available.

### 3.3 Meta-analysis results

#### 3.3.1 Overall sarcopenia prevalence in patients undergoing PD (primary outcome)

**Figure 2:**
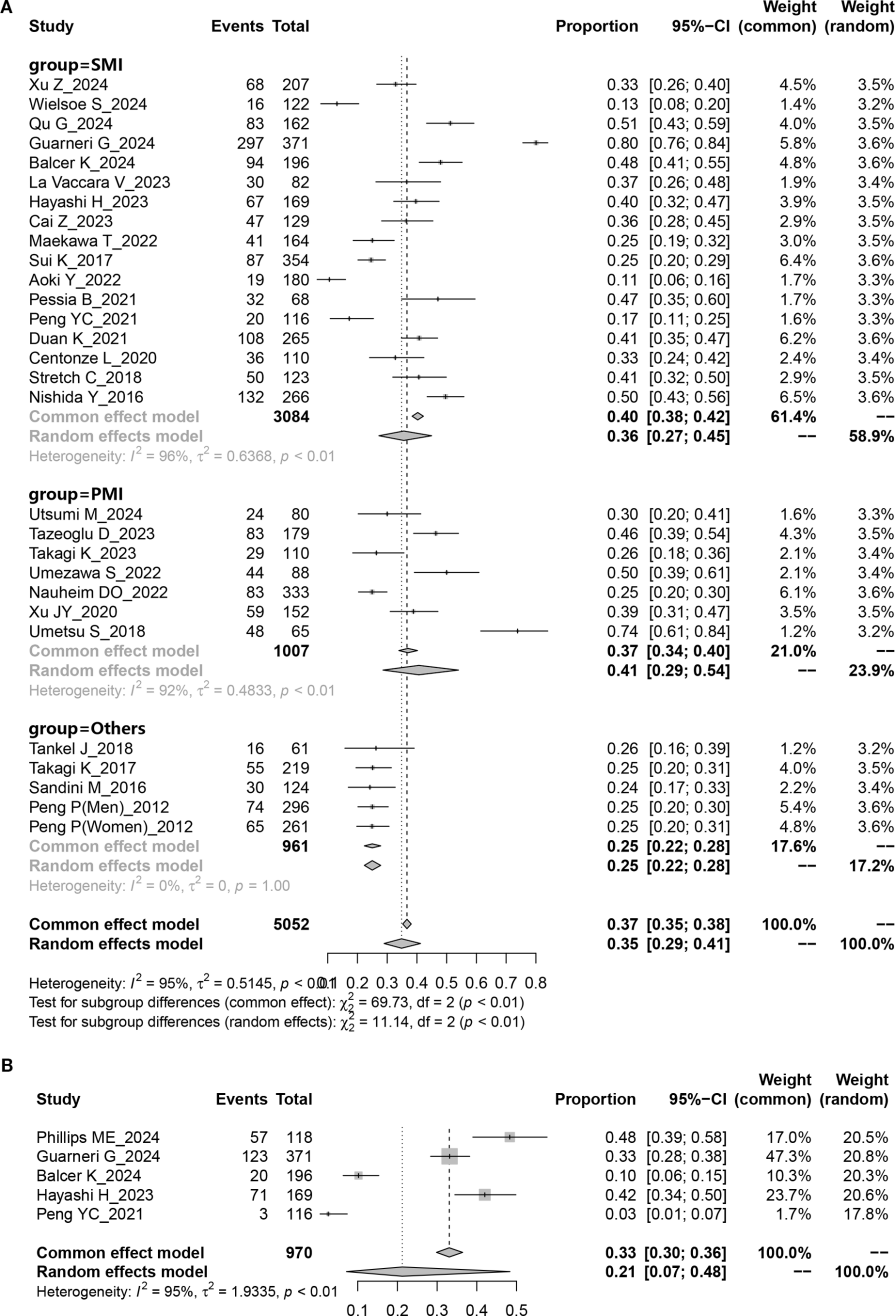
Pooled overall prevalence of sarcopenia and sarcopenic obesity in patients undergoing pancreaticoduodenectomy. **(A)** sarcopenia; **(B)** sarcopenic obesity.

The study indicated a preoperative sarcopenia prevalence of 35% (95% CI = 29%–41%) in patients undergoing PD (**Figure 2A**) with notable heterogeneity (*P <* 0.001; I² = 95%). When SMI was used as the detection indicator, the incidence of sarcopenia was 36% (95% CI = 27%–45%, I² = 96.0%); when PMI was used, the incidence was 41% (95% CI = 29%–54%, I², 92.0%). The results additionally revealed a 36% sarcopenia prevalence in Asian patients undergoing PD (95% CI = 28%–43%, I² = 94.3%, *P <* 0.001), which is lower than the 40% prevalence observed in Caucasian patients undergoing PD (95% CI = 24%–56%, I² = 98.6%, *P <* 0.001) (**Table 2**). Regarding age, the prevalence in patients undergoing PD aged < 65 years (37%, 95% CI = 26%–48%, I² = 98.1%, *P <* 0.001) was lower than those aged > 65 years (39%, 95% CI = 34%–45%, I² = 79.7%, *P <* 0.001) (**Table 2**).

**Table 2:** Comparison of sarcopenia prevalence in patients undergoing pancreaticoduodenectomy regarding age, ethnicity, and sarcopenia assessments.

Subgroup	N	Prevalence (%)	[LL; UL]	P-value	P_heterogeneity_
Overall	29	35	[29;41]	<0.001	<0.001
Sarcopenia measures					
SMI	17	36	[27;45]	<0.001	<0.001
PMI	7	41	[29;54]	<0.001	<0.001
Others	5	25	[22;28]	<0.001	1
Age					
≥65 years	9	39	[26;48]	<0.001	<0.001
<65 years	20	37	[34;45]	<0.001	<0.001
Ethnicity					
Asian	18	36	[28;43]	<0.001	<0.001
Caucasian	11	40	[24;56]	<0.001	<0.001

LL, lower limit of the 95% confidence interval; UL, upper limit of the 95% confidence interval.

##### 3.3.1.1 Publication bias and sensitivity analysis

Funnel plots and Egger’s and Begg’s tests were used to assess potential biases in the literature inclusion process. The funnel plot shows a symmetrical inverted funnel shape. Statistical tests showed no significant bias, with Egger’s and Begg’s tests yielding *P =* 0.583 and *P =* 0.103, respectively. These results suggest the absence of publication bias. A sensitivity analysis was subsequently conducted by sequentially excluding individual studies. No statistically significant variations were observed in the results, thus reinforcing the robustness of our findings (**Supplementary Figures 1A, B**).

##### 3.3.1.2 Overall sarcopenic obesity prevalence in patients undergoing PD (primary outcome)

Five studies provided data on the prevalence of preoperative sarcopenic obesity in patients undergoing PD. The results showed that the overall preoperative sarcopenic obesity prevalence was 21% (95% CI = 0.07%–48%) (**Figure 2B**), with substantial heterogeneity (*P <* 0.001, I^2^ = 95.0%).

##### 3.3.1.3 Publication bias and sensitivity analysis

The funnel plot was symmetrical, and both Egger’s test (*P =* 0.291) and Begg’s test (*P =* 0.260) yielded non-significant results, indicating the absence of publication bias. Sensitivity analysis, excluding one study at a time, showed no significant differences in outcomes, thus further supporting its robustness (**Supplementary Figures 2A, B**).

#### 3.3.2 Secondary outcomes

##### 3.3.2.1 Overall incidence of major complications (CD grade ≥ III)

**Figure 3:**
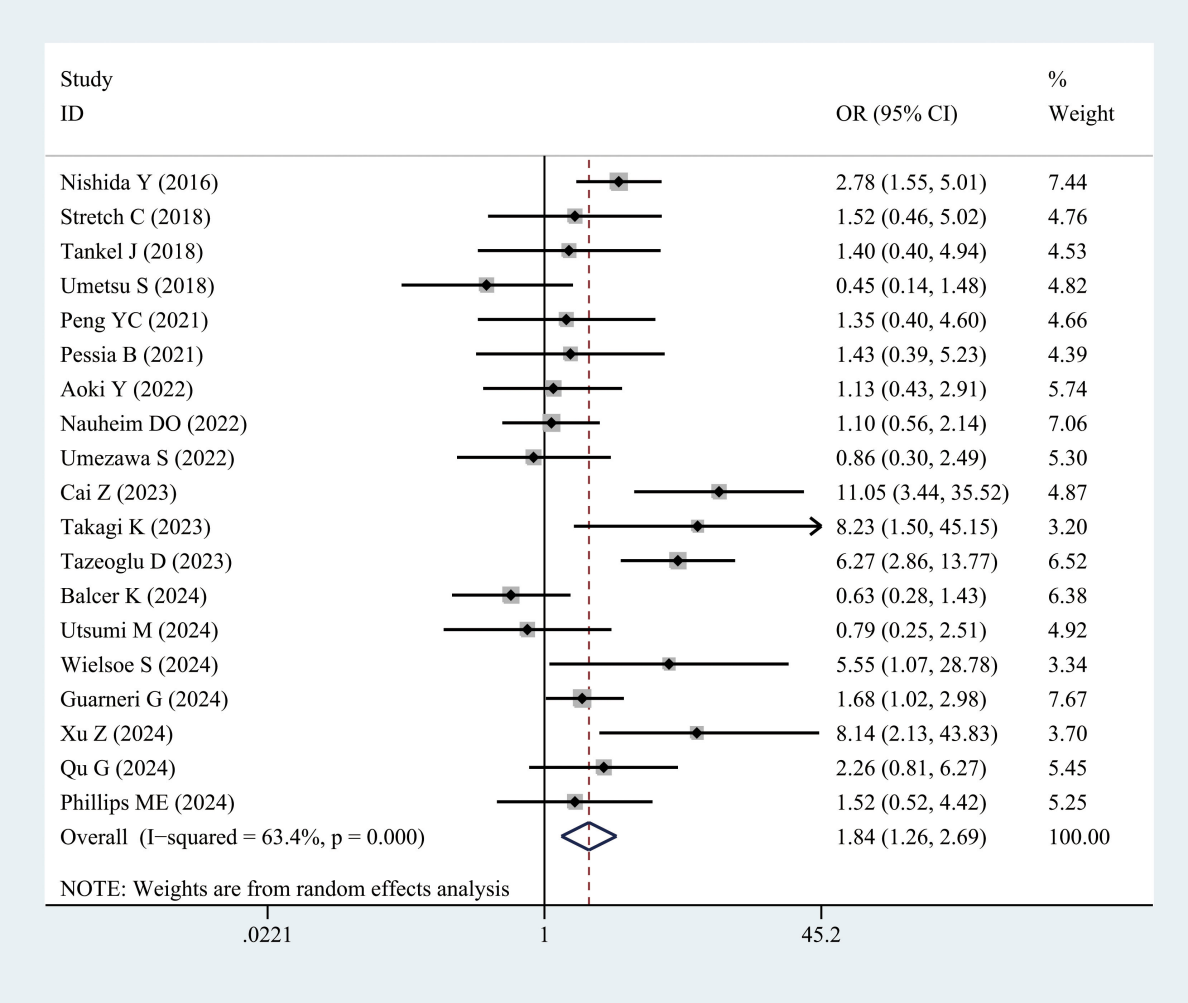
Comparison of the overall rate of major complications (Clavien–Dindo grade ≥ III) between the sarcopenia and non-sarcopenia groups.

Twenty studies reported the incidence of major postoperative complications (CD grade ≥ III) in patients with sarcopenia and matched controls. Most studies indicated a significantly higher incidence of major complications in patients with sarcopenia than in the controls, with an overall rate 1.84 times higher (OR = 1.84, 95% CI = 1.26–2.69, *P =* 0.002) (**Figure 3**, **Table 3**).

**Table 3:** Meta-analyses of secondary outcomes.

Secondary outcomes	Studies	Effect size		P-value	heterogeneity	
		OR (95%CI)	HR (95%CI)		I²(%)	P-value
Overall incidence of major complications (C-D≥III)	20	1.84(1.26,2.69)	–	0.002	64.4	<0.001
Postoperative pancreatic fistula	17	1.47(1.13,1.93)	–	0.004	61.6	<0.001
Postoperative biliary fistula	8	1.53(1.05,2.25)	–	0.028	0	0.528
Mortality rate	11	3.52(2.01,6.19)	–	0.002	31.1	0.151
Disease-free survival	5	–	2.28(1.18,2.88)	<0.001	8.8	0.357
Overall survival	6	–	3.15(2.49,3.98)	<0.001	46.3	0.097

##### 3.3.2.2 POPF

**Figure 4:**
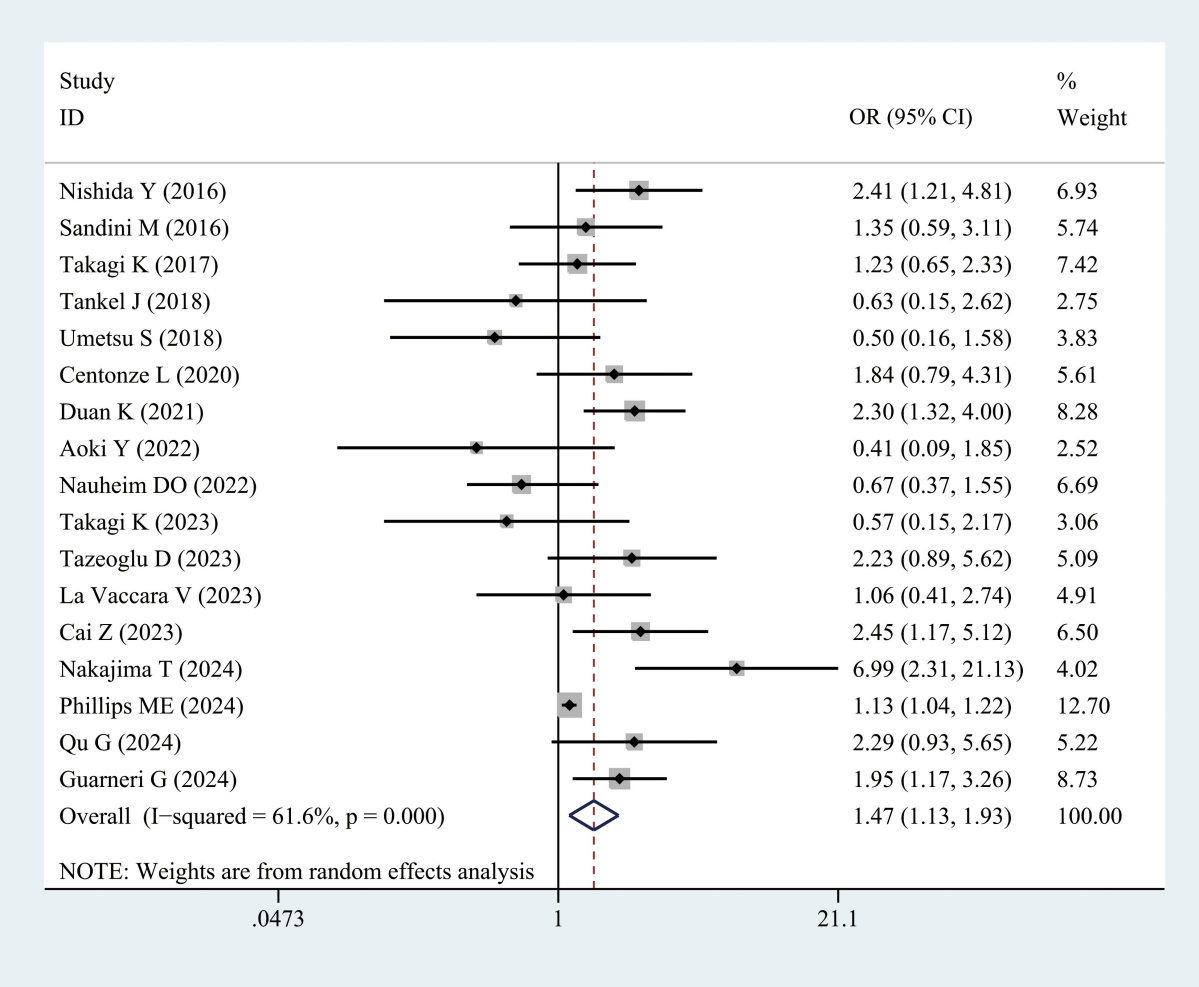
Comparison of the overall rate of postoperative pancreatic fistula between the sarcopenia and non-sarcopenia groups.

Grades B and C fistulas were defined as clinically relevant POPF. Seventeen studies examined the incidence of POPF in patients with sarcopenia compared with the controls. Meta-analysis results revealed a higher incidence of POPF in patients with sarcopenia (OR = 1.47, 95% CI = 1.13–1.93, *P =* 0.004) (**Figure 4**, **Table 3**). POPF is a major complication of PD. Three studies compared the differences in SMI between patients with and without POPF. Nakajima et al. (28) and Hayashi et al. (33) reported that SMI values in patients with POPF were slightly higher than those in patients without POPF. However, Cai et al. (34) demonstrated that the SMI values were lower in patients with POPF, as presented in **Supplementary Figure 3**.

##### 3.3.2.3 POBF

**Figure 5:**
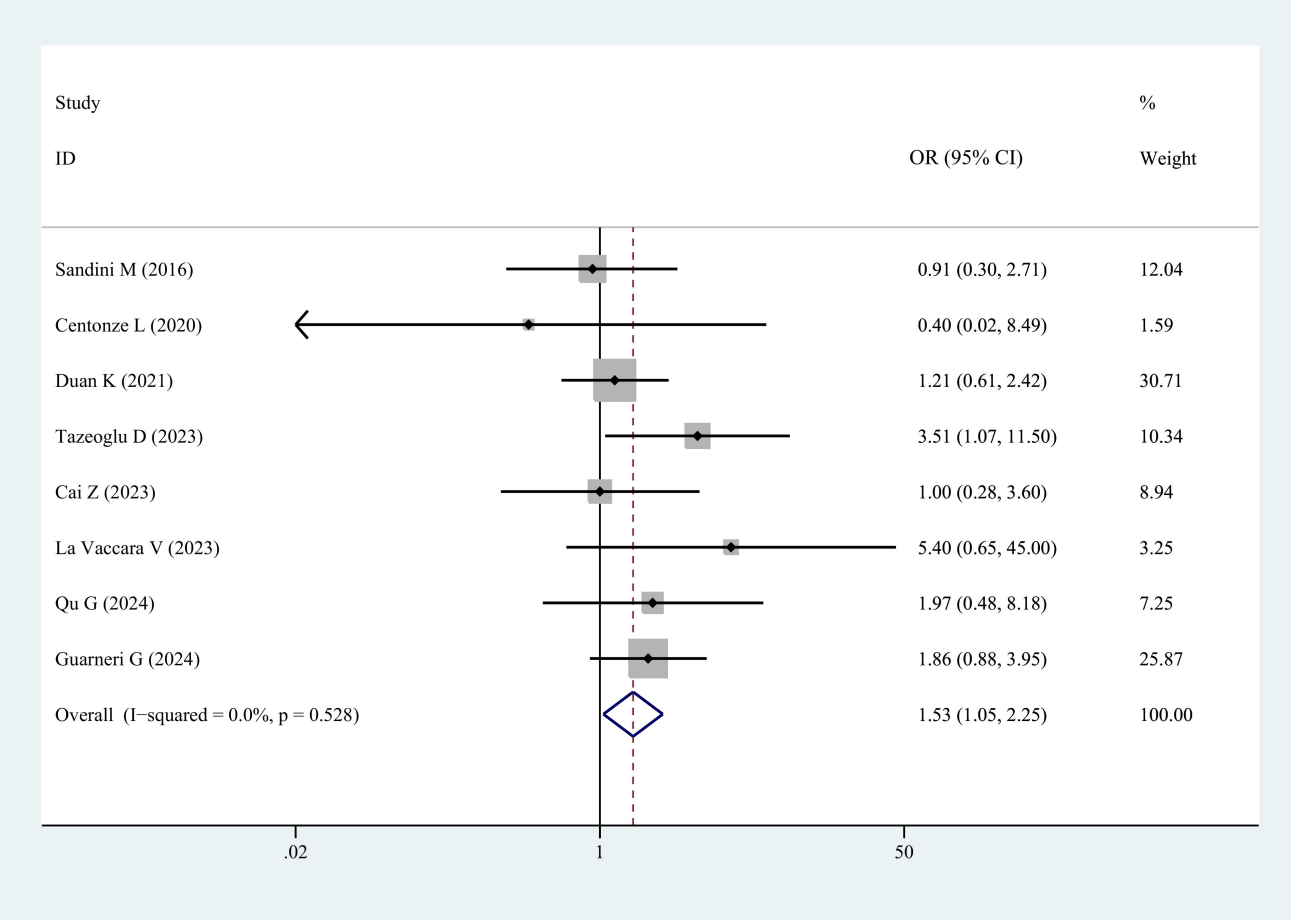
Comparison of the overall rate of postoperative biliary fistula between the sarcopenia and non-sarcopenia groups.

Eight studies reported the incidence of POBF in patients with sarcopenia and controls. The results demonstrated a significantly higher incidence of POBF in patients with sarcopenia (OR = 1.53, 95% CI = 1.05–2.25, *P =* 0.028) (**Figure 5**, **Table 3**).

##### 3.3.2.4 Mortality rate

**Figure 6:**
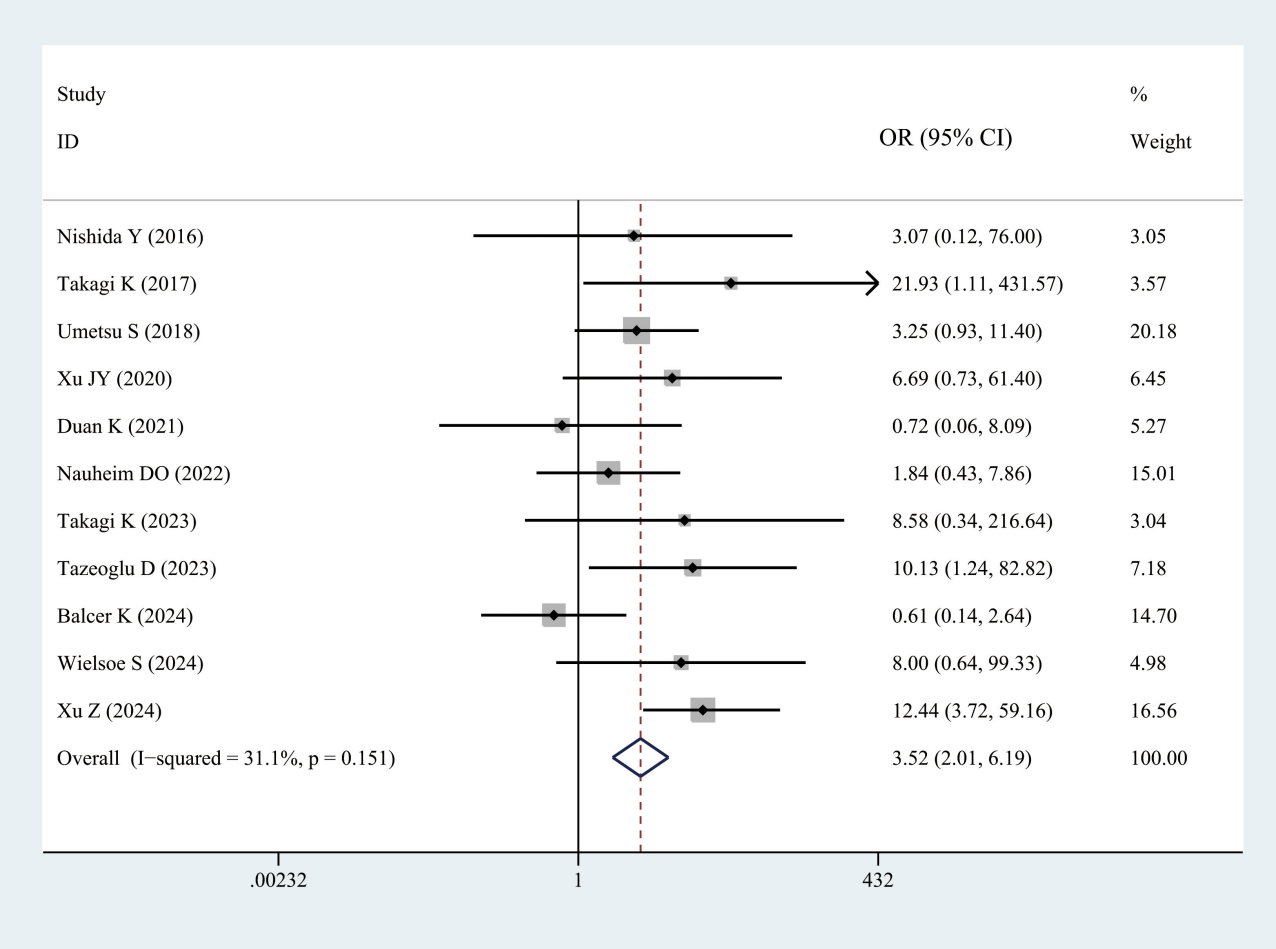
Comparison of the overall mortality rate between the sarcopenia and non-sarcopenia groups.

Eleven studies reported on postoperative mortality. The results demonstrated that patients with sarcopenia exhibited a higher mortality rate (OR = 3.52, 95% CI = 2.01–6.19, *P =* 0.002) (**Figure 6**, **Table 3**).

##### 3.3.2.5 DFS

**Figure 7:**
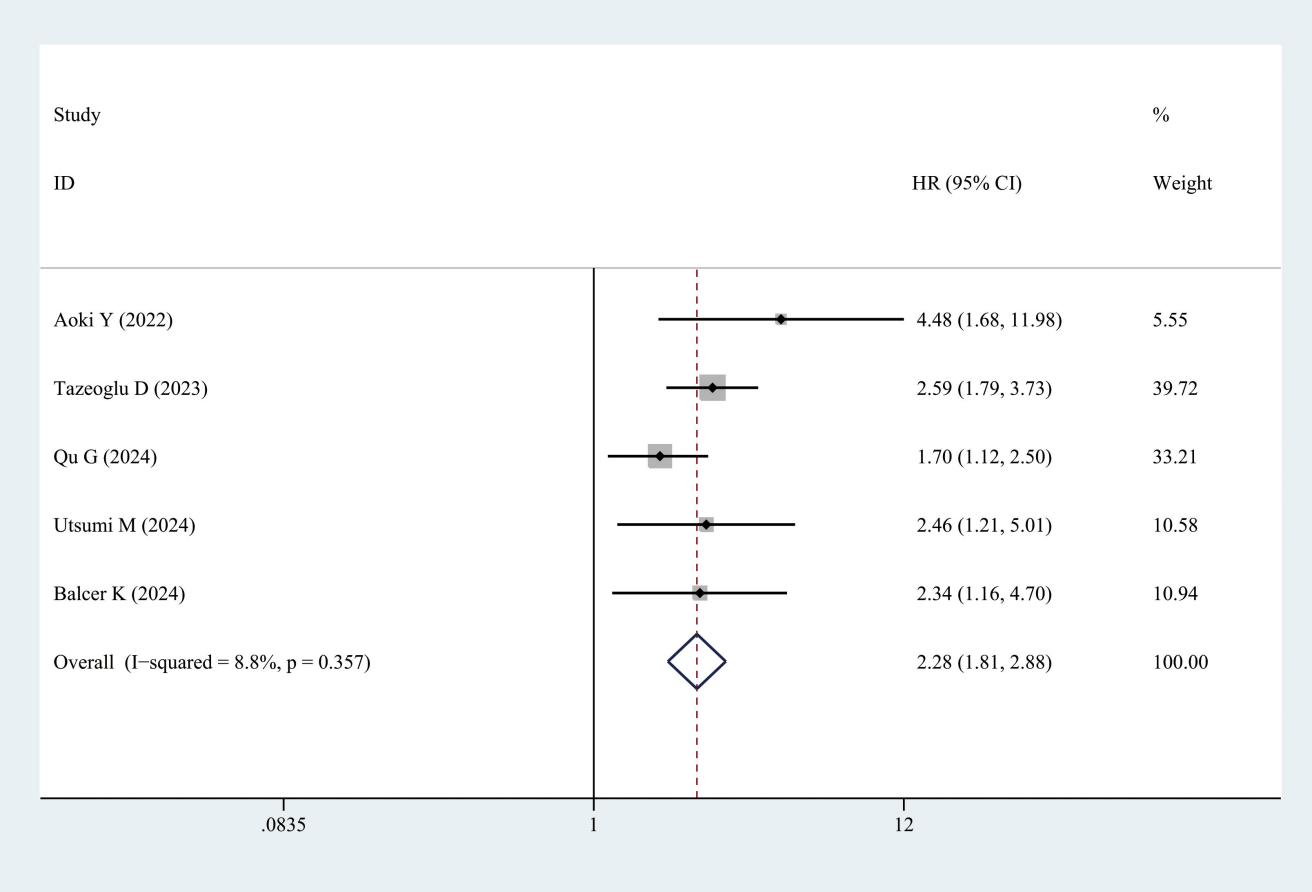
Comparison of the disease-free survival between the sarcopenia and non-sarcopenia groups.

Five studies provided DFS data. Patients with sarcopenia exhibited significantly lower DFS than those without sarcopenia (multivariate analysis: HR = 2.28, 95% CI = 1.18–2.88, *P <* 0.001) (**Figure 7**, **Table 3**).

##### 3.3.2.6 OS

**Figure 8:**
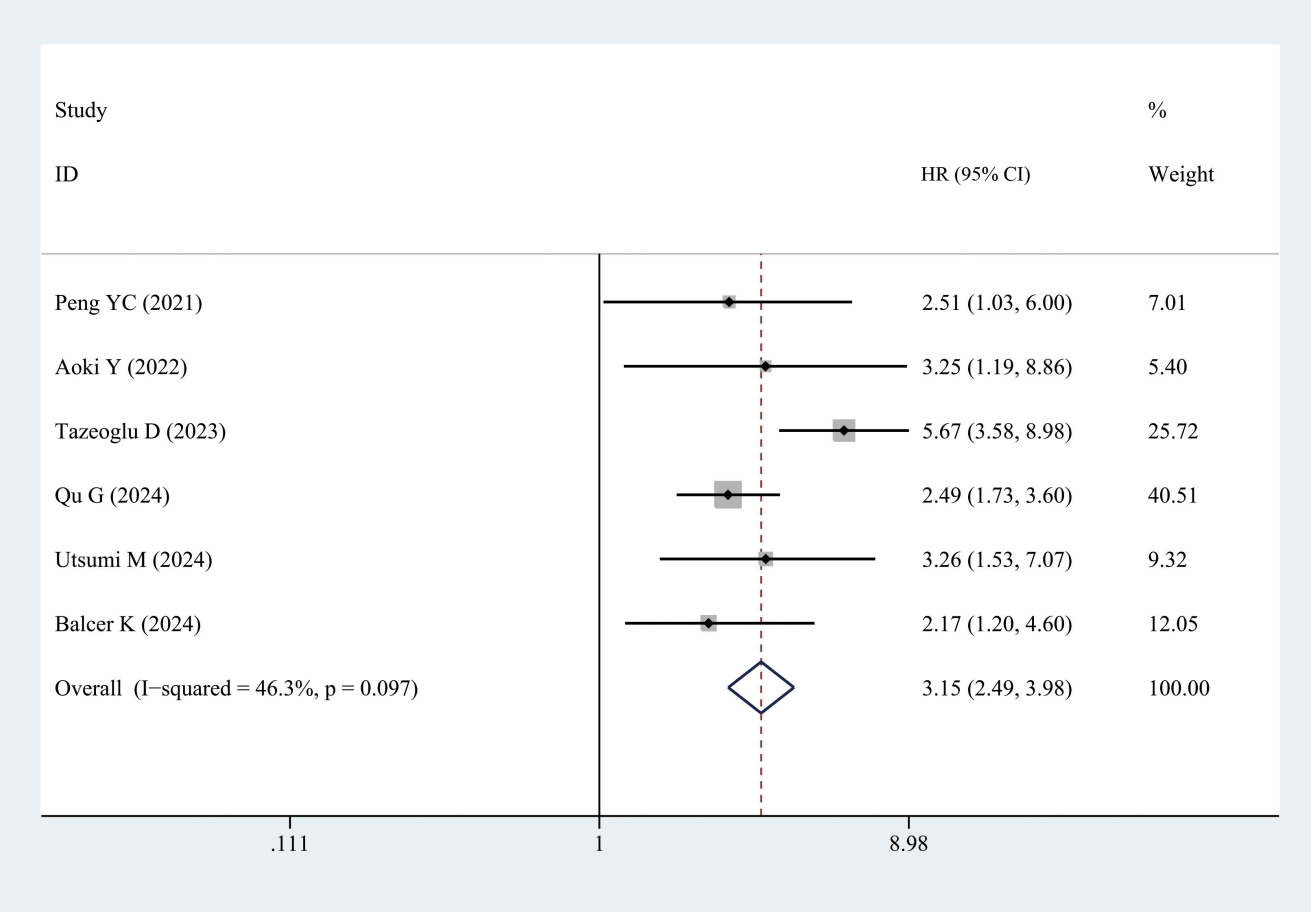
Comparison of the overall survival between the sarcopenia and non-sarcopenia groups.

Six studies reported the OS data. Patients with sarcopenia exhibited significantly worse OS than those without sarcopenia (multivariate analysis: HR = 3.15, 95% CI = 2.49–3.98, *P <* 0.001) (**Figure 8**, **Table 3**).

##### 3.3.2.7 Publication bias

A funnel plot of publication bias across all secondary outcomes is presented in **Supplementary Figure 4**. The evaluation indicated that all the inverted funnel plots were roughly symmetric, thus suggesting a low risk of publication bias.

## 4 Discussion

Sarcopenia is characterized by a gradual decline in both muscle mass and function and is primarily driven by aging, lifestyle factors, and underlying pathological conditions (52). It is prevalent among older adults, with a reported incidence of up to 29%, and contributes significantly to increased disability and mortality (53). The progressive loss of skeletal muscle is a hallmark of sarcopenia, with studies indicating that muscle mass may decrease by as much as 6% annually after middle age (54). Recent studies have indicated a higher incidence of sarcopenia among individuals undergoing surgical interventions, particularly among those with malignancies. The incidence of sarcopenia in patients with liver cancer ranges from 11% to 45% (55). Similarly, sarcopenia affects 33% of patients with cholangiocarcinoma and gallbladder cancer (56), whereas the incidence in patients undergoing surgery for pancreatic cancer varies from 17% to 62% (57). Pancreaticobiliary tumors, which are often associated with obstructive jaundice, malnutrition, compromised intestinal mucosal integrity, and dysbiosis, are key contributors to preoperative sarcopenia (58). Consistent with these reports, the current meta-analysis revealed that 35% of patients undergoing PD presented with sarcopenia preoperatively, whereas 21% exhibited sarcopenic obesity. Consequently, the preoperative assessment of muscle mass and strength in patients undergoing PD is critical because sarcopenia may negatively influence clinical outcomes.

This study examined the prevalence of preoperative comorbid sarcopenia in patients undergoing PD and evaluated the effects of race, age, and diagnostic criteria on sarcopenia rates. These findings indicated significant racial variations in the prevalence of sarcopenia, which were probably due to differences in body composition, lifestyle factors, muscle mass, and strength assessments across geographic populations. Subgroup analysis further revealed a higher prevalence of comorbid sarcopenia in patients aged > 65 years than in those aged < 65 years, thus highlighting the strong association between aging and skeletal muscle loss. Sarcopenia is prevalent among older adults, with epidemiological studies in China reporting rates of 12.9% and 11.2% in community-dwelling men and women, respectively (59). Sarcopenia is characterized by age-related reduction in muscle mass and strength. A Japanese study found that 11.5% of men and 16.7% of women experienced varying degrees of skeletal muscle loss and hypofunction, with prevalence rates exceeding 50% in individuals > 80 years of age (60). Sarcopenia results from a combination of internal and external factors, and aging is a significant contributor. Age-related changes include substantial reductions in skeletal muscle mass, fiber size, strength, and endurance (61). Furthermore, aging is associated with increased systemic inflammation, which may lead to the overactivation of the ubiquitin–proteasome system (UPS). Protein degradation in skeletal muscles is primarily mediated by the UPS and the autophagy–lysosomal system pathways (62). Aging disrupts physiological homeostasis, thus leading to multiorgan dysfunction and frailty, particularly mitochondrial dysfunction; furthermore, aging plays a central role in the onset of sarcopenia (63, 64).

The term “sarcopenia” primarily refers to the loss of muscle mass; however, several international organizations advocate for diagnostic criteria that additionally incorporate reductions in muscle strength and/or physical function alongside muscle mass loss (65). Although this expanded diagnostic framework has gained widespread acceptance in geriatric medicine, cancer research continues to emphasize muscle mass as the primary diagnostic parameter. Most studies included in this analysis relied on a single method for diagnosing sarcopenia, and were predominantly retrospective. Studies that define sarcopenia using only the SMI or PMI lack sufficient rigor. Although CT is considered the gold standard for muscle mass assessment, it does not directly measure muscle strength. Notably, most studies reviewed in this research employed SMI, which was determined by measuring the muscle area on cross-sectional CT scans at the L3 level. However, some studies suggest that skeletal muscle strength and/or physical function may more accurately predict the prognostic relevance of cancer-related sarcopenia, particularly in patients with gastrointestinal tumors (66, 67). Therefore, additional prospective cohort studies are needed to determine whether these markers should be incorporated into sarcopenia diagnostics for patients undergoing PD.

Few comprehensive studies have explored the effect of sarcopenia on the clinical outcomes of patients undergoing PD. To address this gap, a meta-analysis of 30 studies involving 5323 participants was conducted. Six key factors were evaluated, including major complication rates (CD grade ≥ III), pancreatic fistula, biliary fistula, postoperative mortality, DFS, and OS. The analysis revealed a significant association between sarcopenia and several adverse outcomes in patients with sarcopenia compared with those without sarcopenia. Specifically, individuals with sarcopenia exhibited higher rates of major postoperative complications and pancreatic and biliary fistulas, as well as reduced DFS and OS rates. Patients with sarcopenia are often burdened with multiple comorbidities, including osteoporosis, cardiopulmonary insufficiency, and malignancies, and are more prone to malnutrition, skeletal muscle depletion, and fractures (68). Hu et al. (69) reported that sarcopenia was notably linked to diminished lung function and obstructive pulmonary disease, thus suggesting that muscle fiber atrophy associated with sarcopenia could impair respiratory muscle function. Preoperative respiratory insufficiency, prolonged bed rest after surgery, and pain from upper abdominal incisions may further compromise recovery and contribute to complications. In this study, the worse postoperative clinical outcomes and prognoses in patients with sarcopenia could be attributed to preexisting respiratory dysfunction.

Recent studies have demonstrated a strong association between sarcopenia and pancreatic fistula. Nishida et al. (49) evaluated the skeletal muscle area at the L3 level in 266 patients undergoing PD and found that the incidence of POPF was higher in patients with skeletal muscle depletion. Sarcopenia, second only to pancreatic cancer, is a key predictor of POPF complications. Jang et al. (70) similarly identified sarcopenia, particularly sarcopenic obesity, as an independent predictor of POPF complications in patients undergoing PD. This study revealed that the risk of POPF was significantly higher in patients with sarcopenia than in those without sarcopenia. However, whether patients with POPF truly have lower SMI values than those without POPF remains controversial, and this finding may be related to the sample sizes of the included studies. Patients with sarcopenia, particularly those with sarcopenic obesity, often experience systemic malnutrition, which may impair the healing (71). Additionally, a reduction in skeletal muscle and an increase in fat mass, particularly visceral fat, can alter the pancreatic texture, thus complicating pancreaticojejunostomy and increasing the risk of fistula formation (72). Furthermore, visceral fat contributes to surgical complications by releasing proinflammatory cytokines, which may hinder recovery and promote POPF development. Therefore, preoperative sarcopenia assessment should be emphasized in patients undergoing PD, as well as proactive nutritional and exercise interventions, to address malnutrition and muscle wasting. This approach may reduce the incidence of POPF and enhance surgical outcomes.

Sarcopenia significantly affects the perioperative course of PD. A decline in muscle function reduces postoperative mobility, whereas respiratory muscle weakness increases the risk of hypoxia, respiratory complications, and subsequent lung infections (73). Furthermore, as key metabolic organs, the muscles are crucial for the metabolism of proteins, amino acids, and carbohydrates. Loss of muscle mass disrupts the metabolism of these substances, thus predisposing patients to malnutrition before and after surgery. Recent studies (74–76) have further highlighted immune dysfunction, intestinal flora alteration, and elevated inflammatory marker levels (e.g., tumor necrosis factor, interleukin 6, and nuclear factor kappa-light-chain-enhancer of activated B cells) in patients with sarcopenia. These factors collectively impair surgical tolerance and increase perioperative risk. The findings of this meta-analysis emphasized that preoperative comorbid sarcopenia is a significant predictor of poor postoperative outcomes after PD. Studies have demonstrated that hormones secreted by muscle cells inhibit tumor cell growth (77). The reduced expression of these hormones in patients with sarcopenia may contribute to the proliferation and recurrence of tumors post-surgery. Research on patients with gastric cancer, cholangiocarcinoma, and hepatocellular carcinoma undergoing surgery identified sarcopenia as a negative prognostic factor for long-term survival after surgery (51, 78, 79). The clinical relevance of this study lies in its potential to identify patients with sarcopenia through the preoperative screening of patients undergoing PD. Sarcopenia can be evaluated across three domains, namely, muscle strength, muscle mass, and physical status, thus allowing for timely intervention. Nutritional strategies for sarcopenia focus on addressing malnutrition, ensuring adequate protein intake, supplementing with nutrients such as leucine and vitamin D, and modulating the gut microbiota. In addition, personalized exercise regimens, including tailored rehabilitation training at specific times, intensities, and cycles, should be developed based on the patient’s physical condition. A combination of resistance training and aerobic exercises is recommended (80, 81). These strategies offer significant benefits to patients undergoing PD.

Despite these strengths, this study has a few limitations: (1) All included articles are cohort studies and predominantly retrospective, thus necessitating further validation through randomized controlled trials. (2) Only English- and Chinese-language publications were considered, thus potentially introducing language bias and limiting the comprehensiveness of the review. (3) PD is a complicated surgery used to treat both non-cancerous and cancerous conditions in the pancreatic head, periampullary area, and distal common bile duct. The subgroup analysis was not conducted based on disease type. (4) Variations in sarcopenia diagnostic criteria and cutoff values across studies may have influenced the results. (5) POPF is the most common and dreaded complication following PD, with an incidence ranging from 9% to 50% (82–86). The incidence of POBF ranges from 4% to 12% (87–90). However, it can be hypothesized that combined fistulas (POPF/POBF) are associated with higher mortality rates than isolated POPF or POBF. Aghalarov et al. (91) reported that the incidence of POPF/POBF after PD ranges from 1.8% to 7.7%. Analyzing the effect of sarcopenia on POPF/POBF would be highly meaningful. Unfortunately, few studies have reported the simultaneous occurrence of POPF and POBF after PD. The studies included in our review provided separate data on POPF and POBF, making it impossible to extract valid data on combined fistulas from the literature. Therefore, further reports in this emerging research area are anticipated in the near future.

## 5 Conclusion

In conclusion, the prevalence of comorbid sarcopenia among patients undergoing PD prior to surgery was notably elevated, thus significantly influencing postoperative clinical outcomes. Patients undergoing PD with sarcopenia face a higher risk of major complications, clinically relevant POPF and POBF, increased mortality, and exhibit worse DFS and OS. Future research using a more precise definition of sarcopenia is essential to confirm our results. The preoperative screening and evaluation of sarcopenia should be prioritized, with proactive interventions targeting nutrition and exercise in patients undergoing PD to enhance clinical outcomes and overall prognosis.

## Data Availability

The original contributions presented in the study are included in the article/**Supplementary Material**. Further inquiries can be directed to the corresponding author.

## Supplementary material

Supplementary Figure 1 Sensitivity analysis and funnel plots for the prevalence of sarcopenia in patients undergoing pancreaticoduodenectomy. **(A)** Funnel plots of Begg’s test; **(B)** Sensitivity analysis.

Supplementary Figure 2 Sensitivity analysis and funnel plots for the prevalence of sarcopenic obesity in patients undergoing pancreaticoduodenectomy. **(A)** Funnel plots of Begg’s test; **(B)** Sensitivity analysis.

Supplementary Figure 3 Comparison of SMI value between the postoperative pancreatic fistula and non-postoperative biliary fistula groups.

Supplementary Figure 4 Funnel plots for the secondary outcomes. **(A)** Major complications (Clavien–Dindo grade ≥ III); **(B)** Pancreatic fistula; **(C)** Biliary fistula; **(D)** Mortality rate; **(E)** Disease-free survival; **(F)** Overall survival.
