# Supplementary figures and images for: Sarcopenia as a prognostic marker in patients undergoing pancreaticoduodenectomy: an updated meta-analysis
Source: Front Oncol. 2025 Sep 29;15:1656834. doi: 10.3389/fonc.2025.1656834 (PMC12515648; doi:10.3389/fonc.2025.1656834)

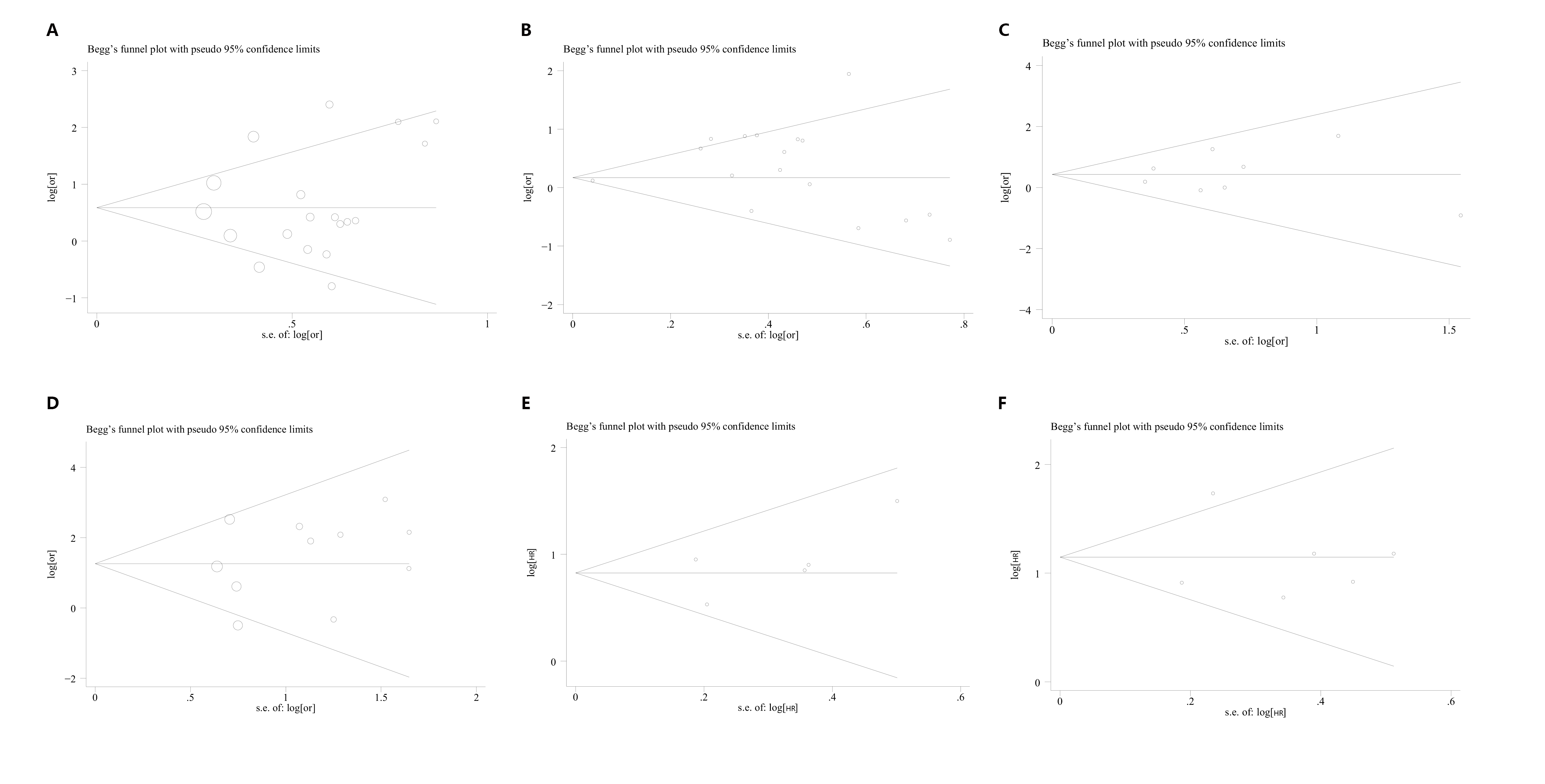

Supplement: Supplementary Figure 1 — Sensitivity analysis and funnel plots for the prevalence of sarcopenia in patients undergoing pancreaticoduodenectomy. (A) Funnel plots of Begg’s test; (B) Sensitivity analysis. [file Image1.tif]

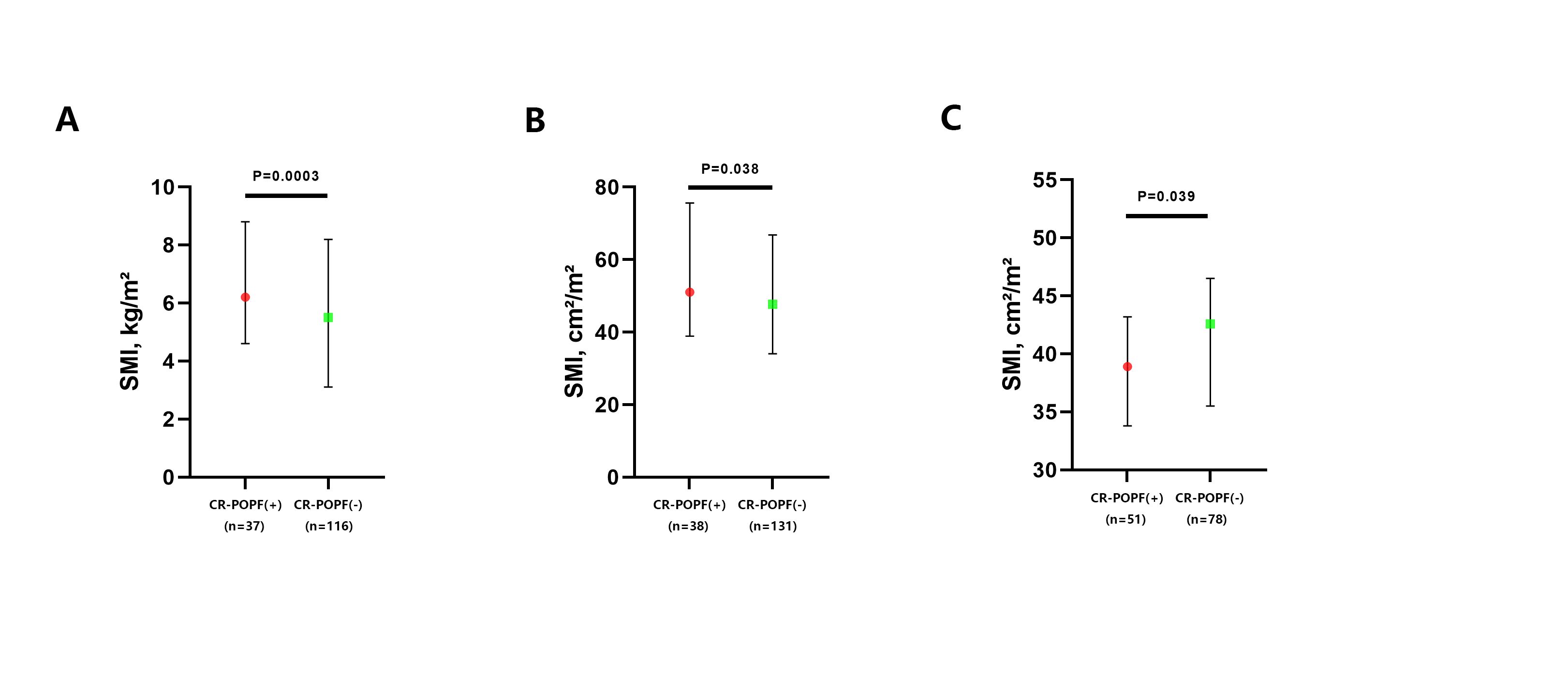

Supplement: Supplementary Figure 2 — Sensitivity analysis and funnel plots for the prevalence of sarcopenic obesity in patients undergoing pancreaticoduodenectomy. (A) Funnel plots of Begg’s test; (B) Sensitivity analysis. [file Image2.tif]

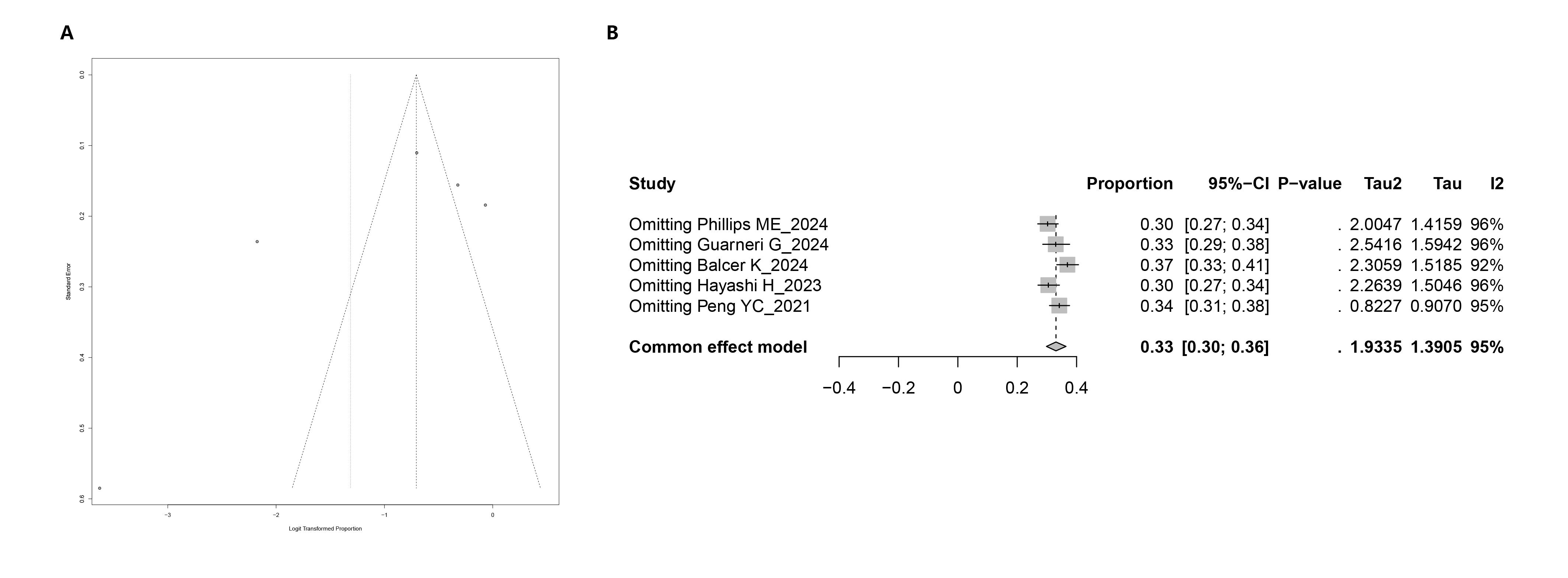

Supplement: Supplementary Figure 3 — Comparison of SMI value between the postoperative pancreatic fistula and non-postoperative biliary fistula groups. [file Image3.tif]

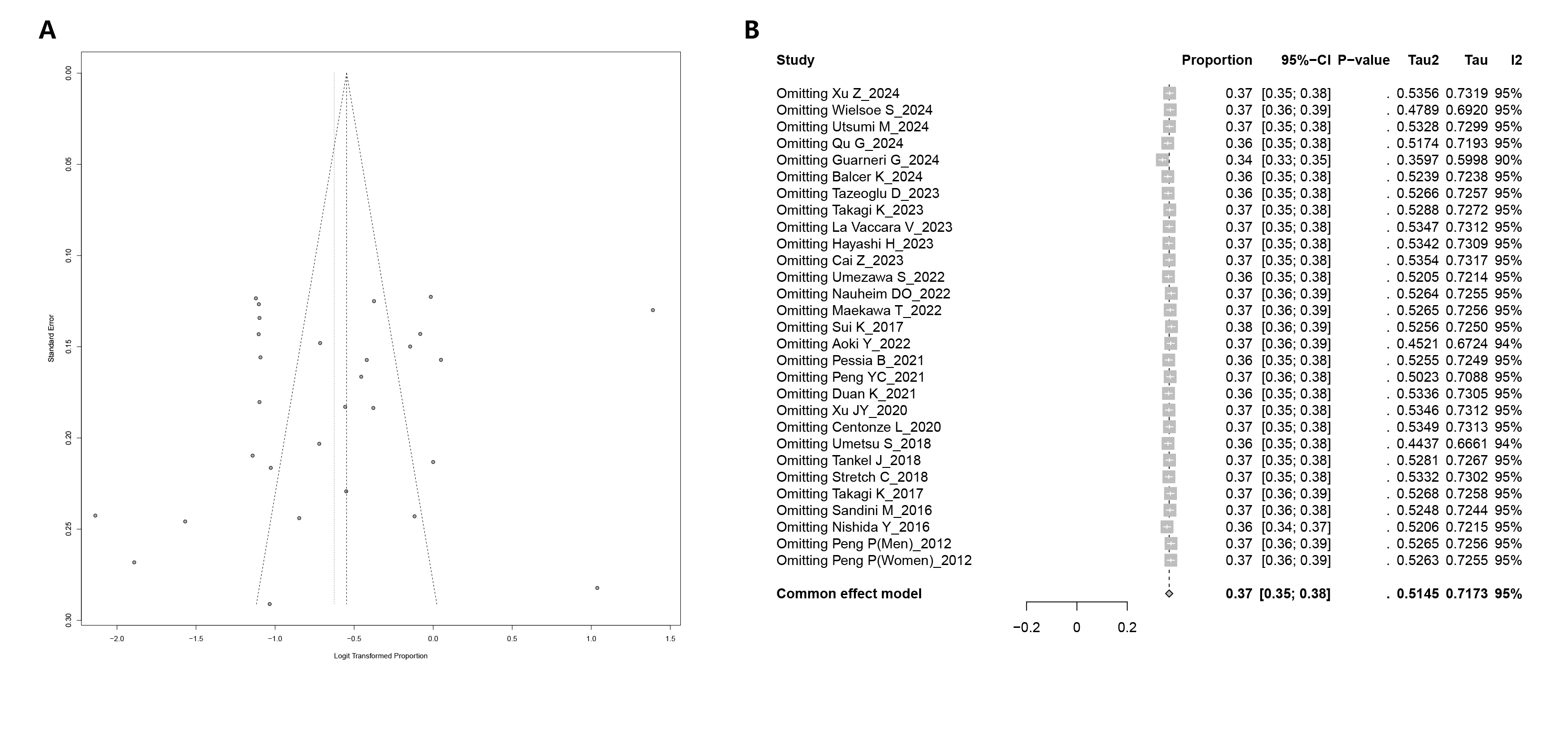

Supplement: Supplementary Figure 4 — Funnel plots for the secondary outcomes. (A) Major complications (Clavien–Dindo grade ≥ III); (B) Pancreatic fistula; (C) Biliary fistula; (D) Mortality rate; (E) Disease-free survival; (F) Overall survival. [file Image4.tif]
