# Supplementary material for: Sarcopenia as a prognostic marker in patients undergoing pancreaticoduodenectomy: an updated meta-analysis
Source: Front Oncol. 2025 Sep 29;15:1656834. doi: 10.3389/fonc.2025.1656834 (PMC12515648; doi:10.3389/fonc.2025.1656834)
Supplement: Supplementary file 5 [file Table1.docx]

**Supplementary Table 1 The checklist items for Joanna Briggs's critical appraisal tool for prevalence studies.**

|  | Yes | No | Unclear | Not applicable |
| --- | --- | --- | --- | --- |
| 1. Was the sample frame appropriate to address the target population? | □ | □ | □ | □ |
| 1. Were study participants sampled in an appropriate way? | □ | □ | □ | □ |
| 1. Was the sample size adequate? | □ | □ | □ | □ |
| 1. Were the study subjects and the setting described in detail? | □ | □ | □ | □ |
| 1. Was the data analysis conducted with sufficient coverage of the identified sample? | □ | □ | □ | □ |
| 1. Were valid methods used for the identification of the condition? | □ | □ | □ | □ |
| 1. Was the condition measured in a standard, reliable way for all participants? | □ | □ | □ | □ |
| 1. Was there appropriate statistical analysis? | □ | □ | □ | □ |
| 1. Was the response rate adequate, and if not, was the low response rate managed appropriately? | □ | □ | □ | □ |
