# Supplementary material for: Sarcopenia as a prognostic marker in patients undergoing pancreaticoduodenectomy: an updated meta-analysis
Source: Front Oncol. 2025 Sep 29;15:1656834. doi: 10.3389/fonc.2025.1656834 (PMC12515648; doi:10.3389/fonc.2025.1656834)
Supplement: Supplementary file 6 [file Table2.docx]

Supplementary Table 2 Demographic characteristics of included studies

| Study | Year | Case | n | Male | Country | Age(years) | Guideline used | sarcopenia measures | Inspection equipment |
| --- | --- | --- | --- | --- | --- | --- | --- | --- | --- |
| Phillips ME | 2024 | 57 | 118 | not  available | UK | 65.1±10.5 | SO was defined as those with a low skeletal muscle index and a  BMI > 30 kg/m^2^ or as a ratio of VFA/SMI with a cut-off of 2.5 m^2^ | Visceral fat area/SMI, Handgrip strength | CT |
| Guarneri G | 2024 | 123 | 371 | 202 | Italy | 60-74 | SO was defined, in line with previous literature,  as a high ratio between  VFA/TAMA, specifically VFA/TAMA ratio greater than 3.2 | Visceral fat area/total abdominal muscle area | CT |
| Balcer K | 2024 | 20 | 196 | 108 | France | 47-67 | Visceral fat area/SMI≥2.5 | Visceral fat area/total abdominal muscle area | CT |
| Hayashi H | 2023 | 71 | 169 | 105 | Japan | 30-92 | The cutoff value sarcopenic obesity were defined corresponding to the maximum sensitivities and  specificities for predicting grade B/C POPF in ROC  curve analysis: the cutoff value was 1.58. | Visceral fat area/SMI | CT |
| Peng YC | 2021 | 3 | 116 | 68 | Taiwan | 66.2±11.9 | Sarcopenic obesity was defined as VAT/TAMA ≥ 2 in sarcopenic patients | VAT/TAMA | CT |

SO: Sarcopenic obesity;

VFA/TAMA: visceral fat area and total abdominal muscle area;

ROC: receiver operating characteristic;

SMI: skeletal muscle index
