# Supplementary material for: Sarcopenia as a prognostic marker in patients undergoing pancreaticoduodenectomy: an updated meta-analysis
Source: Front Oncol. 2025 Sep 29;15:1656834. doi: 10.3389/fonc.2025.1656834 (PMC12515648; doi:10.3389/fonc.2025.1656834)
Supplement: Supplementary file 7 [file Table3.docx]

**Supplementary Table 3 Quality assessment of the included studies.**

| **Author** | **Checklist item** | | | | | | | | | **Score**  **(Out of 9)** |
| --- | --- | --- | --- | --- | --- | --- | --- | --- | --- | --- |
|  | **1** | **2** | **3** | **4** | **5** | **6** | **7** | **8** | **9** |  |
| Xu Z | 1 | 1 | 0 | 1 | 1 | 1 | 1 | 1 | 1 | 8 |
| Wielsoe S | 1 | 1 | 0 | 0 | 1 | 1 | 1 | 1 | 1 | 7 |
| Utsumi M | 1 | 0 | 0 | 1 | 1 | 1 | 1 | 1 | 1 | 7 |
| Qu G | 1 | 0 | 1 | 1 | 1 | 1 | 1 | 1 | 1 | 8 |
| Guarneri G | 1 | 0 | 0 | 0 | 0 | 1 | 1 | 1 | 1 | 5 |
| Balcer K | 1 | 0 | 0 | 1 | 1 | 1 | 1 | 1 | 1 | 7 |
| Tazeoglu D | 1 | 0 | 0 | 1 | 1 | 1 | 1 | 1 | 1 | 7 |
| Takagi K | 1 | 1 | 0 | 1 | 1 | 1 | 1 | 1 | 1 | 8 |
| La Vaccara V | 1 | 0 | 0 | 0 | 0 | 1 | 1 | 1 | 1 | 5 |
| Hayashi H | 1 | 0 | 1 | 0 | 1 | 1 | 1 | 1 | 1 | 7 |
| Cai Z | 1 | 0 | 0 | 0 | 1 | 1 | 1 | 1 | 1 | 6 |
| Umezawa S | 1 | 1 | 0 | 0 | 1 | 1 | 1 | 1 | 1 | 7 |
| Nauheim DO | 1 | 0 | 0 | 0 | 0 | 1 | 1 | 1 | 1 | 5 |
| Maekawa T | 1 | 0 | 0 | 0 | 1 | 1 | 1 | 1 | 1 | 6 |
| Sui K | 1 | 0 | 0 | 0 | 0 | 1 | 1 | 1 | 1 | 5 |
| Aoki Y | 1 | 1 | 0 | 1 | 1 | 1 | 1 | 1 | 1 | 8 |
| Pessia B | 1 | 1 | 1 | 1 | 1 | 1 | 1 | 1 | 1 | 9 |
| Peng YC | 1 | 0 | 1 | 1 | 1 | 1 | 1 | 1 | 1 | 8 |
| Duan K | 1 | 1 | 0 | 1 | 1 | 1 | 1 | 1 | 1 | 8 |
| Xu JY | 1 | 0 | 0 | 0 | 0 | 1 | 1 | 1 | 1 | 5 |
| Centonze L | 1 | 0 | 0 | 1 | 0 | 1 | 1 | 1 | 1 | 6 |
| Umetsu S | 1 | 0 | 0 | 0 | 0 | 1 | 1 | 1 | 1 | 5 |
| Tankel J | 1 | 0 | 0 | 1 | 1 | 1 | 1 | 1 | 1 | 7 |
| Stretch C | 1 | 0 | 0 | 1 | 1 | 1 | 1 | 1 | 1 | 7 |
| Takagi K | 1 | 0 | 0 | 1 | 1 | 1 | 1 | 1 | 1 | 7 |
| Sandini M | 1 | 1 | 1 | 1 | 1 | 1 | 1 | 1 | 1 | 9 |
| Nishida Y | 1 | 0 | 0 | 1 | 1 | 1 | 1 | 1 | 1 | 7 |
| Peng P | 1 | 0 | 0 | 1 | 1 | 1 | 1 | 1 | 1 | 7 |
| Nakajima T | 1 | 1 | 0 | 1 | 1 | 1 | 1 | 1 | 1 | 8 |
| Phillips ME | 1 | 1 | 0 | 0 | 1 | 1 | 1 | 1 | 1 | 7 |
