# Supplementary material for: Sarcopenia as a prognostic marker in patients undergoing pancreaticoduodenectomy: an updated meta-analysis
Source: Front Oncol. 2025 Sep 29;15:1656834. doi: 10.3389/fonc.2025.1656834 (PMC12515648; doi:10.3389/fonc.2025.1656834)
Supplement: Supplementary file 8 [file Table4.docx]

| **Supplementary Table 4. QUIPS risk of bias** | | | | | | | | |
| --- | --- | --- | --- | --- | --- | --- | --- | --- |
| **Author** | **Year** | **Study participation** | **Study attrition** | **Prognostic factor measurement** | **Outcome**  **measurement** | **Study**  **confounding** | **Statistical analysis**  **and reporting** | **Overall rating** |
| Xu Z | 2024 | moderate | low | low | low | low | low | low |
| Wielsoe S | 2024 | low | low | low | low | moderate | moderate | moderate |
| Utsumi M | 2024 | low | low | low | low | moderate | low | moderate |
| Qu G | 2024 | low | low | low | low | low | low | low |
| Guarneri G | 2024 | low | low | low | low | high | low | high |
| Balcer K | 2024 | low | low | low | low | moderate | low | moderate |
| Tazeoglu D | 2023 | low | low | low | low | moderate | low | moderate |
| Takagi K | 2023 | low | low | low | low | low | low | low |
| La Vaccara V | 2023 | low | low | low | low | high | moderate | high |
| Hayashi H | 2023 | low | low | low | low | moderate | low | moderate |
| Cai Z | 2023 | low | low | low | low | moderate | low | moderate |
| Umezawa S | 2022 | low | low | low | low | moderate | low | moderate |
| Nauheim DO | 2022 | low | low | low | low | high | low | high |
| Maekawa T | 2022 | low | low | low | low | high | moderate | high |
| Sui K | 2017 | low | low | low | low | moderate | low | moderate |
| Aoki Y | 2022 | low | low | low | low | moderate | low | moderate |
| Pessia B | 2021 | low | low | low | low | low | low | low |
| Peng YC | 2021 | low | low | low | low | moderate | moderate | moderate |
| Duan K | 2021 | low | low | low | low | low | low | low |
| Xu JY | 2020 | moderate | high | low | low | low | low | high |
| Centonze L | 2020 | low | low | low | low | moderate | low | moderate |
| Umetsu S | 2018 | low | low | low | low | high | moderate | high |
| Tankel J | 2018 | low | low | low | low | moderate | low | moderate |
| Stretch C | 2018 | low | low | low | low | moderate | low | moderate |
| Takagi K | 2017 | low | low | low | low | moderate | low | moderate |
| Sandini M | 2016 | low | low | low | low | low | low | low |
| Nishida Y | 2016 | low | low | low | low | moderate | low | moderate |
| Peng P | 2012 | low | low | low | low | low | low | low |
| Nakajima T | 2024 | low | low | low | low | low | low | low |
| Phillips ME | 2024 | low | low | low | low | moderate | moderate | moderate |
